# “Detection of sedative and antipsychotic in exhumed institutionalized patients: a forensic toxicological case series”

**DOI:** 10.1007/s12024-025-01081-w

**Published:** 2025-09-18

**Authors:** Gianmarco Argentiero, Luca Morini, Marcello Benevento, Laura Ambrosi, Simona Nicolì, Davide Ferorelli, Biagio Solarino

**Affiliations:** 1 Department of Legal Medicine and Forensic Sciences, University of Pavia, Via Forlanini n. 12, Pavia, 27100 Italy; 2 Department of Legal Medicine and Forensic Sciences, University of Bari “Aldo Moro”, Bari, Italy

**Keywords:** Case series, Forensic toxicology, Drugs intoxication, Exhumation, Decomposition, Post-mortem redistribution, Midazolam, Promazine

## Abstract

Post-mortem toxicological analysis in exhumed bodies presents considerable methodological and interpretative difficulties. These limitations become particularly relevant in judicial contexts involving elderly individuals in long-term care, where pharmacological regimens may be deliberately omitted from medical records. This study investigates a series of 15 exhumed cases in which the detection of sedative and antipsychotic drugs, particularly Midazolam and Promazine, raised concerns of potentially unauthorized or inappropriate administration. All cases underwent full forensic autopsy, including external examination and sampling of available biological matrices. Toxicological analyses were performed using GC-MS and LC-MS/MS platforms. Quantitative results were evaluated alongside pathological findings to assess potential contribution to the cause of death. Advanced decomposition affected all cases, severely compromising morphological and histological evaluation. Autopsy findings revealed multiple chronic conditions, consistent with age-related comorbidities. Toxicological screening identified Midazolam, its metabolite α-hydroxy-midazolam, and Promazine in various tissues. However, only one subject had a documented prescription for these agents. In the remaining 14, no therapeutic indication was recorded. All concentrations were interpreted with caution due to decomposition-related artifacts. The interpretation of drug levels in decomposed exhumed bodies is limited by post-mortem redistribution, instability of substances, and the absence of validated reference ranges for degraded matrices. Toxicological positivity, in the absence of medical justification, may support hypotheses of unauthorized administration but cannot by itself establish a causal relationship with death. These findings underscore the need for cautious interpretation and further research on drug behaviour in post-mortem decomposed tissue.

## Introduction

Post-mortem toxicology plays a pivotal role in forensic medicine, particularly when the cause or manner of death is unclear. Its importance becomes even more pronounced in judicial contexts involving potential criminal liability, where forensic conclusions are expected to meet the highest standard of proof to be reported by trial to convict the defendant beyond any reasonable doubt (BARD). However, the trustworthiness of toxicological interpretations is strongly influenced by the quality of the biological matrices examined [1]. In exhumed bodies - often affected by advanced putrefaction and structural degradation - both anatomical-pathological and toxicological assessments are fraught with interpretative limitations [2]. Putrefactive processes can alter the distribution and stability of substances within the body, leading to a phenomenon such as post-mortem redistribution (PMR) [3]– [4], which challenge the reliability of both qualitative and quantitative toxicological analyses [5]. Such analyses become even more challenging when dealing with chronically or terminally ill individuals, often treated with sedative or palliative medications - circumstances that may later prompt judicial authorities to order exhumation in order to clarify the appropriateness or legitimacy of pharmacological management. Exhumation represents one of the most complex procedures in forensic pathology and it is requested by prosecutors when new suspicions arise regarding the cause and manner of death after burial, when the initial investigation is deemed incomplete or inconclusive, or when judicial authorities require further information to confirm or refute criminal responsibility. The examination of exhumed remains is challenging, as it often involves considerable analytical uncertainty due to advanced decomposition, environmental degradation, and the scarcity of viable biological matrices [6]– [7]. These factors can limit the evidentiary value of findings, particularly when forensic toxicology is expected to provide definitive answers regarding exposure, dosage, and potential contribution to death. The detection of xenobiotics in post-mortem toxicological analyses, especially when unsupported by clinical records, raises medico-legal concerns regarding undocumented or even unlawful administration [8]– [9]. In recent years, the forensic community has become increasingly attentive to the pharmacological management of vulnerable populations, especially in long-term care facilities, where limited external oversight may facilitate inappropriate or excessive medication practices. While some cases are attributable to errors in documentation or end-of-life comfort protocols, others may reflect potentially negligent or abusive pharmacological conduct. The fine line between palliative sedation and improper drug use must therefore be rigorously assessed - medically, ethically, and legally - especially when allegations of involuntary intoxication are brought forward [10]. Among the substances frequently used in such settings, midazolam is of particular interest. It is a short-acting benzodiazepine with potent anxiolytic, sedative, hypnotic, and anticonvulsant effects, primarily acting through positive allosteric modulation of GABA-A receptors. Due to its rapid onset and brief duration of action, midazolam is widely used for procedural sedation, seizure control, and palliative sedation in terminal care. However, its administration in elderly or frail individuals carries a high risk of adverse events, including respiratory depression, hypotension, and enhanced central nervous system depression, particularly in the context of polypharmacy. In Italy, midazolam in injectable form is a controlled substance, and every administered dose must be accurately documented in compliance with national regulations [11]. This study presents a case series of 15 exhumed individuals who died while institutionalized in the internal medicine department of a healthcare facility. Post-mortem toxicology revealed the presence of midazolam and/or promazine in various biological matrices. The work aims to discuss the interpretative challenges posed by the detection of these compounds in decomposed remains and to underline the need for a cautious, multidisciplinary forensic approach. This report should be considered as a descriptive case series rather than a statistical or causality-driven investigation.

## Materials and methods

This study included 15 exhumed individuals who died while residing in the internal medicine department of a healthcare facility. The cohort comprised both male and female subjects, aged between 75 and 96 years (mean: 85.5 years), and a post-mortem interval (PMI) ranging from 120 to 194 days, all of whom were affected by chronic, progressive, and often multi-organ pathologies - including cardiovascular, respiratory, metabolic, renal, and neoplastic conditions. All deaths occurred in an institutionalized medical setting under continuous pharmacological management. Exhumations were ordered by the judicial authority following concerns regarding the potential unauthorized administration of sedative or antipsychotic drugs, in the context of a more exhaustive forensic investigation. As exhumation procedures were required, they were performed in compliance with current national mortuary regulations, which stipulate that the opening of sealed zinc coffins must occur under controlled conditions. Accordingly, all coffins were opened in the presence of judicial officers and the forensic team, immediately prior to the external examination. The external inspection aimed to verify the identity of the remains, evaluate the degree of post-mortem preservation, and document any residual medical devices (e.g., feeding tubes, catheters, surgical incisions). Despite the presence of advanced putrefactive changes, the condition of the bodies allowed for the complete execution of both external and internal autoptic procedures. Complete internal autopsies were conducted using standard forensic protocols. Based on preservation status, either the Virchow technique (individual organ dissection) or, in selected cases, the Ghon technique (en bloc organ removal) was adopted [12]. Macroscopic analysis was followed by systematic sampling for histological and toxicological purposes. Tissues were collected from the liver, spleen, kidney, lung, brain, heart, gastrointestinal tract, uterus, prostate and, when present, from neoplastic masses. Samples for histology were fixed in 10% buffered formalin, embedded in paraffin, sectioned at 3–5 μm, and stained with hematoxylin and eosin (H&E) for light microscopy [13]. For toxicological analysis, multiple biological matrices were sampled, including solid organs (liver, spleen, kidney, lung, brain, adipose tissue, and muscle), body fluids when present (cavity blood, pleural and pericardial fluid), and keratinous materials, including (hair). All specimens were retrieved under sterile conditions, placed in appropriate containers, and stored refrigerated or frozen depending on analytical requirements. Toxicological screening and quantification were performed using a combination of gas chromatography–mass spectrometry (GC-MS), liquid chromatography–tandem mass spectrometry (LC-MS/MS), and high-resolution quadrupole time-of-flight mass spectrometry (LC-QTOF). Sample preparation included homogenization, solid-phase extraction, and derivatization where required. All procedures were validated for post-mortem analysis and applied to degraded tissues [14]. Quantitative determinations were conducted using matrix-matched calibration curves and internal standards to ensure analytical reliability despite variability related to decomposition [15]. Furthermore, a broad toxicological screening was performed to assess the potential presence of additional compounds capable of enhancing central nervous system depression. This panel, analyzed via GC-MS and LC-QTOF platforms, included opioids and their metabolites - such as morphine and 6-monoacetylmorphine (6-MAM), antidepressants, antipsychotics, benzodiazepines, barbiturates, drugs of abuse, and new psychoactive substances (NPS).

## Results

**Fig. 1:**
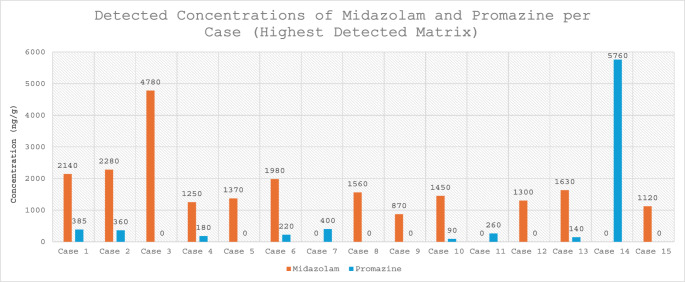
Highest detected concentrations (ng/g or ng/mL) of Midazolam and Promazine per case across analysed biological matrices

A complete overview of all 15 exhumed cases is reported in Table 1. The cohort consisted of eight females and seven males, with an age range from 75 to 96 years (mean: 85.5 years), and a post-mortem interval (PMI) ranging from 120 to 194 days. All individuals were elderly patients with prolonged hospitalization or institutionalization in end-of-life care settings, affected by complex chronic diseases including oncological, cardiovascular, neurodegenerative, and metabolic conditions. At the time of external examination - performed following the opening of the zinc coffin in compliance with national regulations - multiple cadavers presented with classical signs of putrefaction, including epidermolysis, soft tissue liquefaction, and discoloration. In some cases, more atypical features were noted, such as mummification, adipocere formation, corification, and mold growth (black, green, or white) that was extensively distributed on the body surface, particularly the face and thorax [16]– [17]. These conditions significantly hindered the evaluation of possible traumatic injuries, none of which were macroscopically identifiable. Complete internal autopsies were performed in all cases. Although several organs - particularly the spleen, brain, and lungs- were severely affected by autolysis and colliquation, sampling was attempted where feasible. Notably, the natural pathological findings observed at autopsy were coherent with the medical records, which reported terminal neoplastic conditions, chronic ischemic heart disease, hepatic cirrhosis, pulmonary emphysema, and neurodegenerative syndromes. No traumatic lesions were found in any of the examined individuals [18]. Histological samples, taken from the best-preserved organs (liver, kidney, brain, lung), were fixed, processed, and stained with hematoxylin-eosin. Microscopic evaluation confirmed the presence of chronic and degenerative pathological changes, including vascular sclerosis, fibrosis, parenchymal atrophy, and, in several cases, metastatic cancer. Toxicological analysis was performed on all cases using multiple biological matrices including liver, spleen, brain, blood, adipose tissue, muscle, hair and fluid compartments - depending on their preservation status. Each case was positive for at least one pharmacologically active substance. Midazolam and its metabolite α-hydroxy-midazolam were detected in 14 cases, while Promazine was found in 11 cases. The mean concentration of Midazolam in liver samples was 2150 ± 980 ng/g, with a range from 870 to 4780 ng/g, while Promazine showed a mean liver concentration of 1980 ± 1120 ng/g, ranging from 360 to 5760 ng/g. These levels are comparable to those previously reported in exhumation cases with prolonged postmortem interval (PMI) and advanced decomposition, where Midazolam concentrations ranged between 500 and 5000 ng/g in hepatic tissues [2–23]. In this series, liver and brain tissues provided the most reliable detection rates, with Midazolam and Promazine consistently identified in over 90% of samples. In contrast, detection in peripheral blood was sporadic, potentially due to advanced hemolysis and PMR artifact production [19]. Of the 15 cases examined, Midazolam was detected in 93.3% of individuals, while Promazine was identified in 73.3%. Both substances were simultaneously present in 66.7% of cases (ten individuals). Only Midazolam was found in 26.7% (four individuals), and only Promazine in 6.7% (one individual), confirming that at least one of the two drugs was detected in all individuals [20]. Additional compounds detected included morphine (*n* = 5), paracetamol (*n* = 4), citalopram (*n* = 2), fentanyl (*n* = 1), oxycodone (*n* = 1), and amlodipine (*n* = 2), reflecting the complexity of pharmacological regimens in elderly institutionalized patients. Among all cases, Case 2 presented the highest levels of Promazine, with 5760 ng/g in liver and 1560 ng/g in spleen. Case 3 exhibited the highest Midazolam concentration, reaching 4780 ng/g in liver. These and other quantitative results are further analysed in the discussion section and reported in Fig. 1. Moreover, the analytical protocol included specific screening for 6-monoacetylmorphine (6-MAM) to exclude heroin-derived exposure; however, no positive results for this metabolite were observed.

**Table 1:** Summary of demographic, pathological, and toxicological data from 15 exhumed individuals

Case ID	Sex	Age (years)	PMI (days)	Main comorbidities	Histological samples collected	Toxicological matrices analyzed	Analytical techniques used	Drugs detected	Documented prescription
1	M	82	124	Heart failure, Diabetes, Metastatic prostate cancer	Liver, Lung, Kidney, Brain	Liver, Spleen, Brain, Blood	LC-MS/MS, GC-MS	Midazolam, α-hydroxy-midazolam, Promazine	No
2	F	81	144	COPD, Vascular dementia, chronic kidney disease	Liver, Kidney, Lung, Brain, Heart	Liver, Spleen, Fat, Muscle	LC-MS/MS, LC-QTOF	Midazolam, Promazine	No
3	F	96	120	Hypertension, Alzheimer’s disease, Atrial fibrillation	Liver, Kidney, Brain	Liver, Spleen, Brain	GC-MS, LC-QTOF	Midazolam	No
4	M	91	186	Chronic heart disease, Parkinsonism	Liver, Kidney, Lung, Brain	Liver, Spleen, Blood	LC-MS/MS	Midazolam, Promazine	No
5	F	78	135	Diabetes, Osteoporosis, Senile dementia	Liver, Lung, Brain	Liver, Spleen	LC-MS/MS, GC-MS	Midazolam	No
6	F	86	167	Cognitive impairment, Stroke	Liver, Kidney, Lung	Liver, Spleen, Brain	LC-MS/MS, LC-QTOF	Midazolam, Promazine	No
7	M	83	192	Renal failure, Cognitive impairment	Liver, Kidney, Lung	Liver, Brain, Blood	GC-MS	Promazine	No
8	F	81	140	Multiple myeloma, COPD	Liver, Kidney, Brain	Liver, Spleen	LC-QTOF	Midazolam	No
9	F	75	145	Hypertension, Type 2 diabetes, Alzheimer’s disease	Liver, Lung, Brain, Heart	Liver, Spleen, Brain	LC-MS/MS	Midazolam	Yes
10	F	88	129	Chronic kidney disease, Hypertension	Liver, Kidney, Brain	Liver, Brain	LC-MS/MS, GC-MS	Midazolam, Promazine	No
11	M	80	155	Cardiomyopathy, Vascular dementia	Liver, Lung, Brain	Liver, Spleen	LC-QTOF	Promazine	No
12	F	82	194	Diabetes, Alzheimer’s disease	Liver, Kidney, Lung	Liver, Brain	LC-MS/MS	Midazolam	No
13	M	92	173	Stroke, Congestive heart failure	Liver, Lung, Brain, Heart	Liver, Brain, Fat	GC-MS, LC-QTOF	Midazolam, Promazine	No
14	F	93	138	Osteoarthritis, COPD, Hypertension	Liver, Kidney, Lung, Brain	Liver, Spleen	LC-MS/MS	Promazine	No
15	M	80	153	Alzheimer’s disease, Chronic heart disease	Liver, Brain, Kidney	Liver, Brain, Blood	LC-MS/MS, GC-MS	Midazolam	No

## Discussion

Post-mortem toxicology in exhumed individuals presents substantial interpretative challenges, especially when the context involves allegations of inappropriate pharmacological administration in a vulnerable, institutionalized population in an advanced stage of decomposition. The individuals included in this study were elderly, chronically ill, and institutionalized in a long-term care facility where pharmacological treatments were routinely employed. Among the psychoactive drugs commonly administered in such settings, midazolam holds particular relevance due to its widespread use for sedation and palliative care [21]– [22]. Despite its clinical utility, the substance is considered high-risk in geriatric pharmacology and, depending on national regulations, is subject to strict prescribing and recording protocols. Nonetheless, the exhumations were ordered to clarify whether the presence of sedative and antipsychotic agent - specifically Midazolam and/or Promazine - could support the hypothesis of unauthorized or non-therapeutic administration. A direct comparison between toxicological results and clinical records revealed a striking inconsistency [23]: although Midazolam and Promazine were detected in the vast majority of cases, only one individual (Case 9) had a documented prescription for Midazolam, administered in the setting of palliative care. In all other cases, there was no medical indication or traceable documentation to support the administration of either drug, despite their presence in multiple biological matrices. Autopsy findings were consistent with the natural disease profiles reported in the medical records. The observed pathological changes were in line with the expected terminal conditions of elderly, institutionalized patients. No traumatic injuries were identified in any subject, nor were there autoptic findings that, in isolation, would strongly support an exogenous cause of death. However, the role of the drugs detected cannot be dismissed. While the toxicological analyses provided quantitative concentrations for Midazolam, Promazine, and other substances, these values must be interpreted with caution. Post-mortem redistribution (PMR), tissue degradation, and drug instability in decomposed samples introduce significant variability and uncertainty into any quantitative assessment. In the absence of premortem samples and well-defined reference thresholds for decomposed tissues, the correlation between concentration and toxicity becomes intrinsically unreliable. Furthermore, the exclusive inclusion of elderly individuals constitutes a limitation of the present case series. Age-related alterations in drug metabolism, organ function, and tissue composition may have influenced both antemortem pharmacokinetics and postmortem redistribution, thereby introducing additional variability that must be taken into account in the interpretation of the toxicological results. In the absence of premortem samples and well-defined reference thresholds for decomposed tissues, the correlation between concentration and toxicity becomes intrinsically unreliable. Nonetheless, the application of validated techniques such as GC-MS and LC-MS/MS remains a cornerstone of forensic toxicology, enabling the detection of target substances even in degraded or limited matrices. This analytical capability plays a crucial role in supporting forensic interpretation, particularly when addressing complex cases involving decomposed remains. In such cases, even an apparently elevated drug concentration cannot, by itself, substantiate a conclusion of intoxication or causality in death. Nonetheless, the detection of non-prescribed pharmacologically active substances in all but one of the subjects cannot be dismissed as coincidental or incidental. While it is not possible to state with certainty whether these drugs directly contributed to death, the evidence strongly supports the occurrence of unauthorized drug administration. Given the documented absence of therapeutic indications and the consistent detection of drugs with sedative and antipsychotic activity - substances that, by law, are subject to controlled prescription and tracking - the hypothesis of illicit administration appears not only plausible, but likely. These findings underscore the importance of interpreting toxicological results within a multidisciplinary forensic-medical framework, incorporating clinical documentation, pathological evidence, and awareness of post-mortem artifacts. They also emphasize the need for additional experimental studies to elucidate the behaviour of specific substances in decomposed matrices, thereby enhancing the forensic reliability of such analyses in exhumation cases.

## Conclusion

This case series illustrates the challenges and limitations of post-mortem toxicological interpretation in exhumed and decomposed individuals, particularly when addressing forensic questions in criminal proceedings. Although quantitative results were obtained for several substances, including Midazolam and Promazine, their interpretation was significantly hampered by post-mortem changes including redistribution and tissue degradation. Despite these limitations, the consistent detection of pharmacologically active compounds, most of which were not prescribed, clearly indicates that drug administration occurred. While it remains uncertain whether these substances directly contributed to the cause of death in each case, the absence of therapeutic justification, combined with their detection in multiple tissues, raises serious medico-legal concerns. Ultimately, this study reinforces the need to integrate toxicological data within a multidisciplinary medico-legal assessment, and to exercise great caution when interpreting post-mortem drug concentrations in exhumed or decomposed bodies. Further research on drug behaviour in decomposed matrices is essential to improve forensic reliability in similar cases.
